# First experiences with a surgery supporting computer system in regard to education, efficiency and complications

**DOI:** 10.1016/j.sopen.2023.11.005

**Published:** 2023-11-21

**Authors:** Isabell Baumann, Alexander Böhringer, Raffael Cintean, Florian Gebhard, Peter H. Richter, Konrad Schütze, Alexander Eickhoff

**Affiliations:** Department of Traumatology, Hand-, Plastic-, and Reconstructive Surgery, Center of Surgery, University of Ulm, Germany

**Keywords:** Education in trauma surgery, Surgical supporting computer system, optimizing surgical processes

## Abstract

**Purpose:**

Since October 2019 a computer software named SPM (surgical process manager) is used in a Level I Trauma center. Workflows were developed for distinct surgical procedures (distal radius fractures, proximal femur fractures, ankle fractures, proximal humerus fractures and vertebral fractures). In addition, these workflows were separated in a shortened „Expert“-versions for consultants and a more detailed „Learner“- versions for residents.

This investigation was intended to show, if and what kind of benefits in regard to efficiency (incision to suture and suture to incision time), education and complications a surgery supporting software can bring.

**Methods:**

SPM was used in 90 cases during October 2019 to June 2022. A control trial with 108 patients was developed, including patients of the same age, having same kind of injuries, receiving the same surgery technique without using the SPM.

The software was installed on the computer in the operation room, projected on head monitors and operated by a foot pedal. Complications could also be documented using the pedal.

Groups were divided in surgical procedures and fracture type, qualification of the surgeon, complications and surgery time. Surgery times were taken from the hospital computer system (SAP IS-H). A statistical analysis was performed by using the chi square and Fischer exact test with significance set at a *P* value <0.05.

**Results:**

In 51 cases the software was used for the distal radius (control group 54 patients), in 20 cases for Weber fractures (control group 21 patients), in 9 cases for the proximal femur (control group 19 patients), in 5 cases for vertebral fractures (control group 7 patients) and in 5 cases for the proximal humerus (control group 6 patients).

Time from incision to closure was significant higher in the intervention group (49 vs 42 min, p- value 0,018) and wasn't significant lower in the “expert” group, fixing radius and ancle fractures (39 to 46 min, *p* value 0,186).

Comparing the SPM and control group concerning closing to incision time, no difference could be observed (56 to 58,5 min, *p* value 0,828).

The greatest time deviation between „Learners“und „Experts“was observed in reduction and fixation (*p* value 0,006) in ankle fractures. The “Expert“group also needed less time for the approach (p value 0,008) in case of distal radius fractures.

Unexpected events were more often observed in the intervention group (5,5 vs 3,7 %).

**Conclusions:**

A surgery supporting computer system might be a good tool for detecting and optimizing workflows in the operation room and for improving and analyzing the training of residents and surgical assistants.

In addition, it offers the opportunity to document intraoperative complications. However, a saving of time wasn't observed in this study. Further investigations with bigger number of cases and a longer follow-up are necessary to proof these findings statistically.

## Highlights

• No significant time deviation was observed when trainees performed surgery.
• Using a surgery supporting system increases the possibility of preparing for interventions.
• The software offers the opportunity to analyze each step including complications of pervious interventions.
• The systems helps to standardize the education of young surgeons for typical procedures.

## Introduction

Nowadays even in the operation room digitalization gets more and more common. The main goal is to improve patient safety and outcome by controlling and optimizing different processes of the operation. Unstructured processes may lead to a an overuse of instruments, a longer operation time and might increase the employees dissatisfaction [1,2]. In hospital resources are very valuable, therefore every effort should be made to reduce time and costs.

To improve and digitalize different tasks in the operating room the surgical process institute (SPI) developed the surgical process manager (SPM). SPM is a digital platform with the aim to support the whole team of the operation roam (OR). Surgical procedures are deposited step by step. Monitors in the OR show the current and the following steps of the operation. After surgery a report can be generated and the team performance concerning surgery time is documented. Furthermore every step of the operation can be evaluate and weakness analyzed and further trained. SPM shell keep the quality of supply on a high standard independent of time, place and surgery team [3]. In addition to that the software can be used for the documentation of incidents. Searching for literature shows the obviously lack of experience in trauma surgery.

Early results in paranasal sinus surgery have shown that surgery time and the number of instruments can be reduced by using SPM [4]. Graichen et al. showed that in case of knee replacements, surgery time, number of instruments and number of complication can be reduced as well [5].

Especially in teaching and university hospitals the education of young surgeons should be a central point of the daily business. The literature shows an increasing of surgery time of 20 % and more when a resident is performing surgery, depending on the procedure and the year of the traineeship [6]. Studies investigating the influence of SPM on the education are unknown. A hypothesis of this study is that SPM can at least improve the structure of education and may reduce the difference of surgery time compared to consultants. The procedures were divided into steps and these steps were analyzed concerning degree of education over time.

This investigation was intended to show, if a surgery supporting software can influence the occurence of complications, the efficiency of the OR team(surgery time, turn over time) and the quality of education.

## Material and methods

In cooperation with the AO (Arbeitsgemeinschaft für Osteosynthesefragen) in total 6 workflows were developed. The meaning of workflow is that every step of the operation is explained, including necessary instruments. Workflows are available for plating of distal fibula fractures, plating of distal radius fractures, plating of proximal humerus fractures, nailing of pertrochanteric fractures and stabilization of vertebral fractures.

In case of dislocated type A1 fractures and B1 fractures of the distal fibula SPM was used. If a simple third pipe plate wasn't enough for fixation, SPM wasn't applicated.

Type A, B and C fractures of the distal radius according to the AO classification were included. Indirect reduction was performed by using a volar locking plate (Synthes 2,4 mm). Patients were excluded when additional reduction techniques or additional fixation was required.

In case of proximal humerus fractures also type A,B and C injuries were included, provided that a delta split could be used in a beach chair position and a fixation with a single locking plate was sufficient (Synthes, Philos).

Type A1, A2 and A3 fractures of the proximal femur were included on condition that closed reduction on the traction table and the application of a short nail (240 mm or 235 mm) was possible. For intramedullary fixation PFNA (proximal femur nail antirotation, Synthes) or TFNA (trochanteric fixation nail advanced, Synthes) was used.

Spine fractures (type A and B) were included when only a dorsal stabilization with 4 pedicle screws was necessary (Viper, Synthes). Patients were excluded when a laminectomy or a ventral stabilization was required.

SPM consists of 3 programs. The SPI designer is used for the development of the workflows, the SPI player displays the workflows on the monitors and SPI Insights offers the opportunity to analyze the procedures, including learning curves, displaying the surgery time of every single surgeon over time. In this study the SPI player is the most important software. Depending on the injury workflows can be divided in 5 to 8 phases and these phases are separated into steps. The steps display the current status of the operation telling the surgeon what to do and telling the surgical assistants which instruments are needed. Also animations can be recorded showing the assembly of the instruments. The two following steps are also shown on the right side of the monitor, supporting the team to be prepared for the rest of the procedure. The surgeon or the assistant changes to the next step by operating the foot pedal. In this study the foot pedal was used by the surgeon. Worklflows for “learners” and less detailed workflows for “experts” were developed to increase the surgeons compliance.

**Fig. 1:**
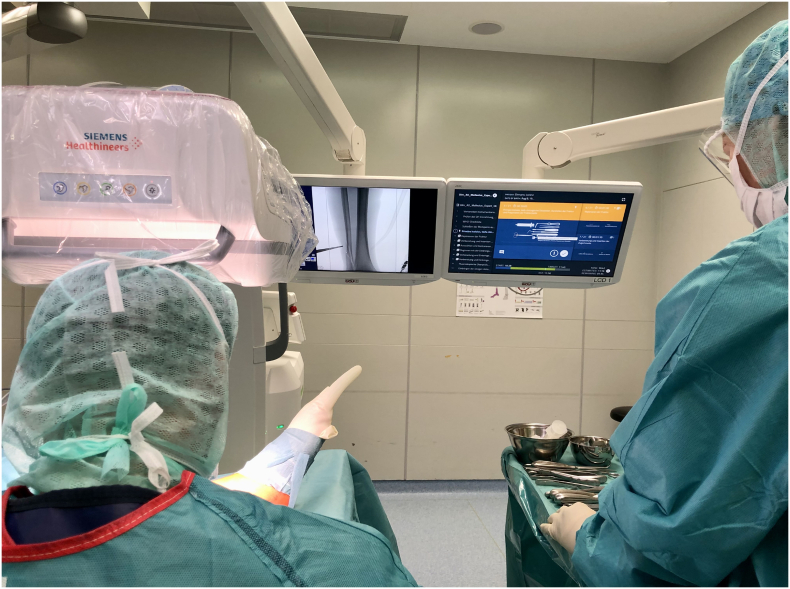
SPI player displays the workflow on the head monitors.

**Fig. 2:**
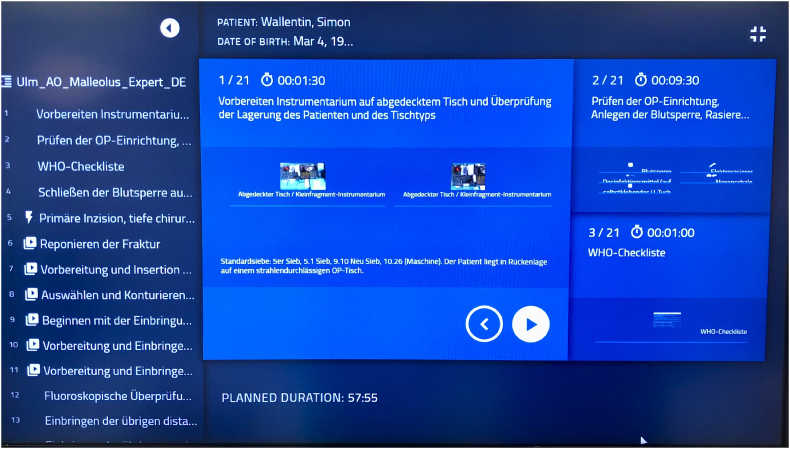
Screenshot of the user interface (first step of distal fibula plating) showing the instrument table in the middle and the two following steps of the procedure on the right sight.

The software was installed on the computer in the operation room, projected on head monitors and operated by a foot pedal (Fig. 1, Fig. 2).

If a complication occurs during the procedure, the right pedal has to be pushed and a list of potential incidents is shown. The appropriate option can be chosen by using the right pedal and has to be confirmed with the left pedal. Complications and comments for each step can also be added after surgery. In the last phase of the workflow x ray time and the AO classification of the fracture were documented.

In total 197 patients were part of the study. All interventions were performed according to the standards of the department independent if SPM was used. The computer supporting system was applied in 90 cases coincidentally. Retrospectively a matching cohort was evaluated as an control group. Matching parameters included age, type of injuries according to the AO classification, surgery technique and surgeons experience, meaning patients of the control group were treated by the same surgeons than patients of the SPI group. If these factors were met, patients were included in the control group. Overall, there were 4 residents, 4 consultants and the chief of the department involved in the study. Groups were divided in surgical procedures and fracture type, experience of the surgeon, complications and surgery time. Surgery time was taken from the hospitals computer system (SAP IS-H).Suture to incision time was evaluated as well as a measure of the efficiency after and before surgery. A statistical analysis was performed by using the chi square and Fischer exact test with significance set at a *P* value <0.05.

## Results

In 51 cases the software was used for the distal radius, in 20 cases for Weber fractures, in 9 cases for the proximal femur, in 5 cases for vertebral fractures and in 5 cases for the proximal humerus.

The control group consists of 54 distal radius fractures, 21 distal fibula fractures, 19 proximal femur fractures, 7 vertebral fractures and 6 proximal humerus fractures.

The average age of the intervention group was 66 years whereas the average age of the control group was 64 years. The group consists of 131 female and 66 male patients.

Observing the intervention group, 61 patients were treated by consultants, whereas in 29 cases surgery were done by residents. In the control group 40 patients were treated by residents.

Table 1 shows on the Y axis the number of interventions. On the x axis the results are separated in the SPI and control group depending on the level of education (blue = trainee, orange = consultant).

**Table 1:**
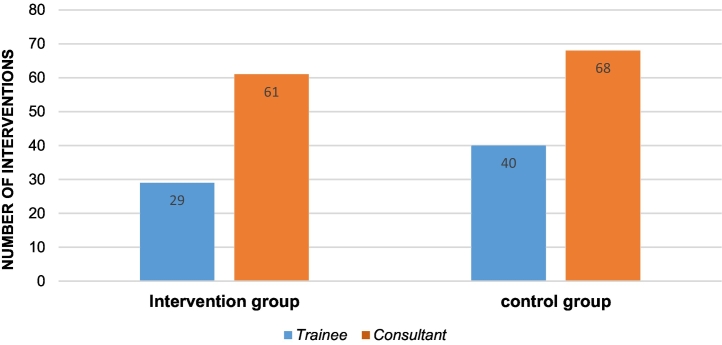
Interventions with and without using SPI, depending on the level of education.

Time from incision to closure was significant higher in the intervention group (49 vs 42 min, p- value 0,018). Table 2 shows the incision to suture time on the Y axis and the two groups (SPI and control group) on the X axis.

**Table 2:**
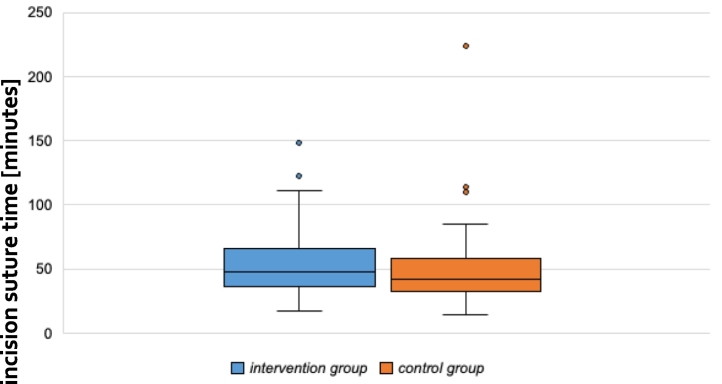
Incision to suture time divided in intervention and control group.

There was no significant time deviation in fixing radius fractures between “experts” and “learners” in the SPM group (39 vs 46 min, *p* value 0,186). Table 3 shows on the Y axis the incision to suture time of the SPI group and on the X axis if fixing of the distal radius with a plate was performed by a consultant or a trainee.

**Table 3:**
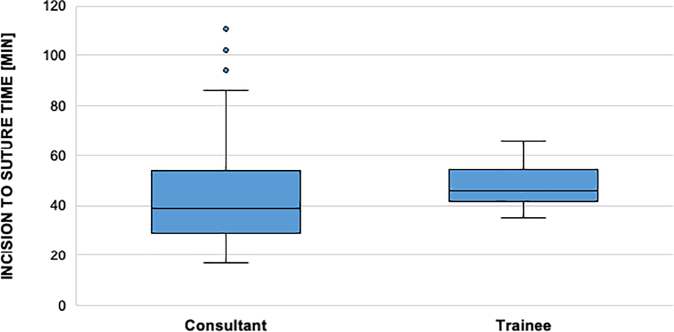
SPI group incision to suture time in fixing distal radius fractures depending on the level of education.

Comparing the control and the intervention group the same pattern can be seen. In the control group no statistical difference of the incision to suture can be observed between trainee and consultant (43 vs 37 min, *p* value 0,061). Table 4 shows on the Y axis the incision to suture time of the control group and on the X axis if fixing of the distal radius with a plate was performed by a consultant or a trainee.

**Table 4:**
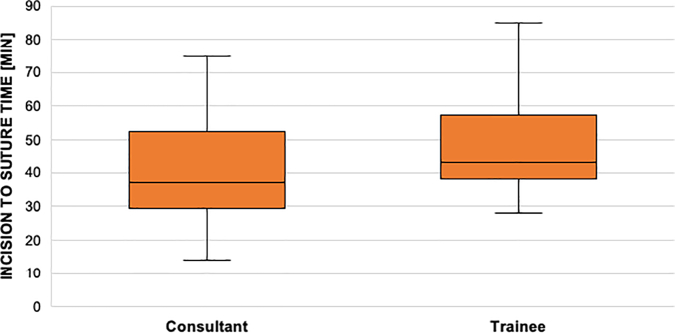
Incision to suture time of the control group in treating distal radius fractures depending on the surgeons level of education.

Examinating the duration of different surgery steps depending on the level of surgeons education the biggest difference is seen in the approach of distal radius fractures (7,8 vs. 11,15 min, *p* value 0,008, Table 5) and the reduction of distal fibula fractures (16,9 vs 24,1 min, p value 0,006). In Table 5 the duration of the approach in minutes is displayed on the Y axis whereas the X axis is separated in consultant and trainee.

**Table 5:**
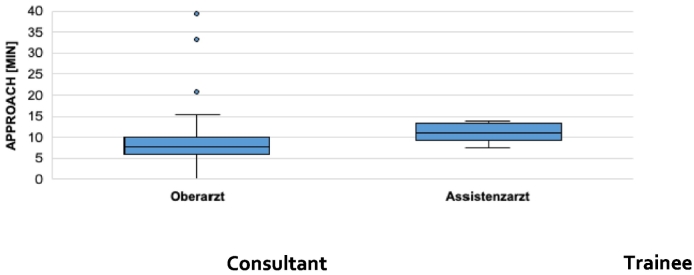
Duration of the surgery step “approach” of distal radius fractures depending on the surgeons level of education.

In Table 6 the duration of the reduction in minutes is displayed on the Y axis whereas the X axis is separated in consultant and trainee.

**Table 6:**
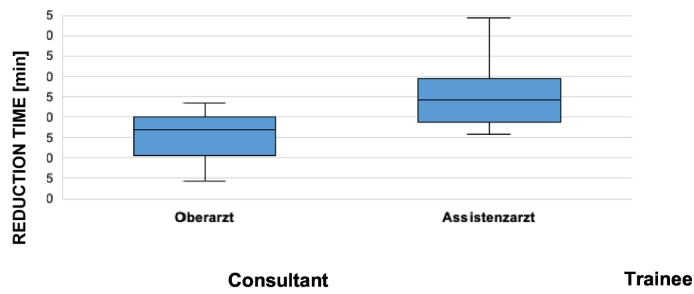
Duration of the surgery step “reduction” in malleolus fractures depending on the surgeons level of education.

In addition to the incision to suture time, suture to incision was evaluated as well. The median time in the SPM group was 56 min, whereas 58,5 min were measured in the control group. No significant difference could be observed (*p* value 0,828). In Table 7 the Y axis includes the suture to incision time in minutes and the X axis is separated in SPI and control group.

**Table 7:**
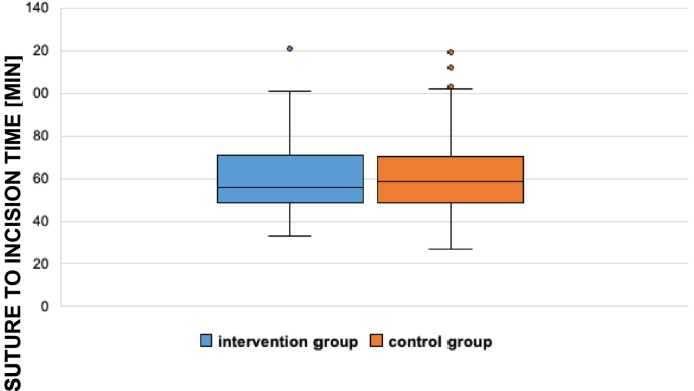
Suture to incision time divided in SPI and control group.

Also complications which occurred during the procedures were documented by the SPM. In total 4 times a lost of reduction was documented and one instrument failure. In the SPI group the complication rate was 5,5 %, whereas 3,7 % were noticed in the control group (p value 0,14). In this group in 4 cases a lost of reduction occurred.

## Discussion

This examination was intended to show what kind of influence a surgery supporting computer software (SPM) may have on the efficiency, on the education and on intraoperative complications. A control group was created.

To evaluate the efficiency of the processes temporal aspects like incision to suture time and suture to incision time were collected. Incision to suture time was significantly enlarged by using SPM (49 vs. 42 min, *p* value 0,018). No significant differences could be seen concerning suture to incision time, showing 56 min in the SPM group and 58,5 min in the control group. There is a limited evidence in the literature showing different results. In comparison to this investigation others showed in case of paranasal sinus surgery shorter surgery time and a reduction of time variance with increasing time of use [4]. The comparisons of these findings is of course quite difficult because of the different study design and different settings, having more standardized procedures in the paranasal sinus surgery. In line with our results Lahmann et al. used SPM for implanting total hip prosthesis and showed no decreasing of the surgery time. Still, Lahmann described a shortened stav in the recovery room, an decreasing number of postoperative anemia and a shortened stay in hospital [7]. In 2023 297 patients underwent a retrospective case control study after knee joint replacement with the result that SPM didn't influence the incidence of an acute haemorrhagic anemia, but reduces the hospital stay, the changeover time and the time in the recovery room significantly [8]. Length of stay in the recovery room and in the hospital wasn't mentioned in this study, but the authors haven't any explanations for a reduction of time in the recovery room and a shortened hospital stay by using SPM. Perhaps other processes in hospital were optimized explaining these findings. An explanation for the even longer surgery time in the SPM group in this investigation could be that at least in the beginning it was unusual to follow the steps displayed on the monitor and the handling of the system was unknown as well. One solution could be that the foot paddle is operated by the assistant and not by the surgeon. Supporting knee arthroplasties SPM was used by Gaichen et al. showing that surgery time can be reduced about 20 % and variance can be reduced about 8 % [5]. Responsible for the different results to this investigation could be that also orthopedic surgery is much more standardized then trauma surgery. In regard to process times (suture to incision time) the evidence in literature is very low as well. The time saving of 2,5 min in this investigation wasn't significant. Lahmann et al. described a reduction of process time (time spending in hospital and in the recovery room) but the total turnover time in the operation room was independent on using SPM [7]. A reason for this could be that other disciplines of the OR (anesthesiologists, anesthesia nurses, surgical assistants) don't participate in a surgery supporting computer system.

A tool like SPM may also be helpful to support the education of young surgeons and surgical assistants. Even before surgery you have the possibility to train the procedures including the instruments step by step. With SPI insights surgeons have also the possibility to get informations about their performance (e.g. in regard to time and complications), displayed on a kind of diagram. In this study the incision to suture time of trainees by using SPM was longer than in the consultant group (46 vs. 39 min) without any significance. In the control group a similar result was found (43 vs. 37 min, *p* value 0,061), meaning that in this investigation the usage of the SPM hadn't any influence on the surgery time independent if it was operated by a trainee or a consultant. In regard to education, SPM and process time no study is known searching for a correlation. Shukla et al. analyzed >400 operations concerning incision to suture time of consultants and trainees [9]. Distal radius fractures, shaft fractures of the radius, shaft fractures of the femur, shaft fractures of the tibia and malleolus fractures were included. Consultants were in total 33 min faster than trainees, so the difference was higher than in this study. One explanation is that Shukla included only surgeons in the third year of their training, whereas the surgeons in this study already passed the first half of the residency. Pförringer et al. showed a time saving of 19,5 % when consultant are performing surgery [6]. These results are in line with the results of this study (time saving about 15 %).

SPM also offers the opportunity to analyze single steps of the operation. A significant difference of time between trainee and consultant is seen by the approach of distal radius fractures and the reduction of distal fibula fractures. Providing these informations is very valuable to focus the education of the trainees. It can be summarized that gadgets like SPM are helpful for education. However, the most important and irreplaceable tool to train surgeons, not at least for legal reasons, is still the experienced surgeon.

Also unforeseeable events can be documented with SPM. By pushing the right pedal a list is displayed on the monitor and you can choose the appropriate complication. In total 5 of these unexpected events occurred in 90 Patients (5,5 %). In 4 cases a loss of reduction and one instrument failure was documented. In the control group 4 events were evaluated, in all cases a loss of reduction. The comparison to the intervention group is difficult, because complications of the control group are only recorded in the operation report. Due to this low number of complications no significant difference between the control and the intervention group and between consultants and trainees could be observed. All the observed complications in this study weren't serious, meaning that the loss of reduction was noticed and the reduction was repeated. So they didn't influence the patients outcome. There are only a few studies looking for intraoperative complications in trauma surgery, in none of them SPM was used for documentation. In most cases intra- and postoperative complications were mentioned [10,11]. Platz and Hyman analyzed 8000 patients regarding intraoperative complications. In 1,5 % of all operations complications occurred [12]. In 13 % these complications weren't documented in the operation report or in the discharge papers. These results are in line with this study, seeing that complications which are documented in the SPM weren't found in the surgery report and complication which were mentioned in the surgery report weren't seen in the SPM. This finding has to draw the attention that the documentation of complications has to be improved in general. On the other hand the possibility of documenting complications in the SPM shell help to define so called critical events. This definition is useful by drawing the hole op teams attention on these steps. Therefore these steps are displayed in different colors on the OR monitors. According to this surgeons are able to find out steps which are challenging like in our case loss of reduction in distal radius fractures. The SPM offers the possibility to create the surgery report and shell be able to trigger the order process in the future. These two features offer the prospect to reduce the workload. In summary it can be said, that a surgery supporting computer system increases the standardization of an OR and the hole team may benefit because of more foreseeable steps and less personal preferences of the surgeon.

This study has some limitations. A big limitation is the quite low number of cases, including different types of procedures resulting in many smaller groups making a comparison more difficult. Another limitation is the retrospective design of the investigation. The control group was built coincidental according to the intervention group. It means that patients were included when type of injury and type of surgery were the same than In the intervention group. Confounding factors weren't mentioned.

## Conclusion

The investigation shows the functions and the potential of computer supporting systems to analyze, optimize and facilitate workflows. First experiences with 90 patients couldn't show a reduction of surgery or turnover time. Besides this SPM offers the opportunity to standardize procedures and to support the education of trainees and surgical assistants. In future these systems are indispensable and the range of features has to be improved to reduce working time and optimize processes. Limitations of this study are the low number of patients and the retrospective design. Further investigations should also address post operative complications by including longer follow up periods.

## Author contributions

Isabell Baumann was the main person who collected data and wrote the manuscript. Alexander Eickhoff developed the theory, wrote the manuscript and collected data as well. Alexander Eickhoff and Florian Gebhard supervised the project. Alexander Böhringer, Raffael Cintean and Peter H Richter collected Data as well. Konrad Schütze verified the analytic methods. All authors discussed the results and contributed the final manuscript.

## CRediT authorship contribution statement

This retrospective chart review study involving human participants was in accordance with the ethical standards of the institutional and national research committee and with the 1964 Helsinki Declaration and its later amendments or comparable ethical standards. The Human Investigation Committee (IRB) of the University of Ulm approved this study.

## Funding

No author is affiliated to any of the supporting companies or received or will receive any form of payment related to this study.

## Declaration of competing interest

The authors declare that there is no conflict of interest. No company had influence in the collection of data or contributed to or had influence on the conception, design, analysis and writing of the study. No further funding was received.
